# [Retracted] Cardioprotective effect of epigallocatechin‑3‑gallate against myocardial infarction in hypercholesterolemic rats

**DOI:** 10.3892/etm.2024.12396

**Published:** 2024-01-22

**Authors:** Wei Zhong, Xiao-Dong Huan, Qian Cao, Jun Yang

Exp Ther Med 9:405–410, 2015; DOI: 10.3892/etm.2014.2135

Following the publication of the above paper, a concerned reader drew to our attention that comparing the histological data shown in Fig. 3 on p. 409 across the four panels led to the identification of several strikingly similar patternings and groupings of the cells, such that data from the same original source(s) may have been used in the various panels to portray the results of supposedly differently performed experiments.

Following an internal enquiry, the Editor of Experimental and Therapeutic Medicine has been able to verify the claims made by the interested reader; therefore, in view of the abovementioned potential anomalies that have been identified, and owing to a lack of overall confidence in the presented data, the Editor has decided that the paper should be retracted from the Journal. The authors were asked for an explanation to account for these concerns, but the Editorial Office did not receive a reply. The Editor apologizes to the readership of the Journal for any inconvenience caused.

